# The Mediating Effect of Smoking on the Association between Income and Dementia among Japanese Older People

**DOI:** 10.31662/jmaj.2025-0018

**Published:** 2025-06-13

**Authors:** Satomi Shimada, Yusuke Matsuyama, Katsunori Kondo, Jun Aida

**Affiliations:** 1 Department of Dental Public Health, Graduate School of Medical and Dental Sciences, Institute of Science Tokyo, Tokyo, Japan; 2 Center for Preventive Medical Sciences, Chiba University, Chiba, Japan; 3 Institute for Health Economics and Policy, Association for Health Economics Research and Social Insurance and Welfare, Tokyo, Japan

**Keywords:** longitudinal study, Cox proportional hazard analysis, causal mediation analysis, health inequalities

## Abstract

**Introduction:**

Health inequalities in dementia have been reported. Smoking is a risk factor for dementia and is disproportionately distributed in marginalized populations. This study examined the mediating effect of smoking on the association between income and dementia among older Japanese people.

**Methods:**

This longitudinal study was based on the Japan Gerontological Evaluation Study from 2010 (response rate = 64.3%) to 2019 (follow-up rate = 98.5%). A total of 44,083 participants independent in daily life were analyzed (mean age: 73.7; women 53.2%). The outcome variable was the incidence of dementia between 2010 and 2019, and the explanatory variable was equivalent income measured in 2010. Causal mediation analyses with a Cox proportional hazard model were performed to evaluate the mediating effect of smoking status in 2010. Multiple imputation was performed for the missing data.

**Results:**

Among the participants, the incidence rates of dementia were 16.2% for men and 18.2% for women. Low income was associated with the incidence of dementia (total effect in excess relative risk, ERR [95% confidence interval (CI)]: 0.095 [0.032-0.157] in overall participants, 0.102 [0.011-0.192] for men, 0.082 [−0.003 to 0.168] for women). Causal mediation analyses showed that smoking mediated the association between income and dementia (natural indirect effect in ERR [95% CI]: 0.007 [0.004-0.011] for overall participants, 0.007 [0.002-0.013] for men, and 0.005 [0.001-0.009] for women). The proportions of the mediating effect were 7.7% for all participants, 7.3% for men, and 6.4% for women.

**Conclusions:**

Our results showed that smoking partially explained the association between income and dementia. There is a possibility that smoking cessation may contribute to reducing health inequalities in dementia.

## Introduction

Dementia is a progressive cognitive impairment that leads to a decline in daily function, mobility, and independence. Worldwide, more than 57 million people have dementia, and the number is expected to increase to 153 million by 2050 ^(1)^. In addition, the total global economic cost of dementia in 2019 was 1,313.4 billion US$, which corresponds to US $23,796 per person living with dementia ^(2)^. Therefore, dementia is a global public health concern in aging societies.

Low socioeconomic status (SES) is known to be a key risk factor for dementia. Among the main components of adulthood SES (i.e., education, occupation, and income ^(3), (4)^), a low educational background is associated with dementia ^(1), (5)^. A previous study showed that improvements in academic standards in the U.S. contributed to reducing socioeconomic inequalities in dementia from 2000 to 2016 ^(6)^. However, education is generally determined in young adulthood ^(3)^ and is difficult to modify at older ages. Therefore, studies focusing on aspects of SES that are modifiable in older age, such as income, would provide insight into public health strategies to reduce health inequalities in dementia in the current aging society. Some studies have reported that the risk of dementia onset is higher in people with lower income than in those with higher income ^(4), (7)^. For example, individuals with higher household incomes can receive earlier medical treatment, and the severity of dementia is milder than in those with lower incomes ^(7)^.

Smoking is a substantial public health problem and caused 8.71 million deaths in 2019 ^(8)^. Smoking accelerates the decline of cognitive function and age-related thinning of the brain cortex, which leads to dementia ^(9)^. While the prevalence of smoking is decreasing worldwide, socioeconomic inequalities in smoking remain remarkable ^(10), (11)^. For example, low income leads to psychological stress due to a negative social environment ^(12)^, and psychological stress is known as a significant risk factor for smoking ^(13)^. In addition, smoking cessation behavior is known to spread through social networks, and smoking status may differ by social class. Therefore, smoking could mediate the association between SES and dementia. A previous study with 32 years of follow-up reported that smoking in middle age mediated 16% of the association between socioeconomic position and future dementia onset ^(14)^. However, it remains unclear whether smoking status in older age mediates the association between SES in older adults and the incidence of dementia. This longitudinal study aimed to elucidate the mediating effect of smoking on the association between income and the incidence of dementia among older Japanese adults.

## Materials and Methods

### Study settings

**Figure 1.**
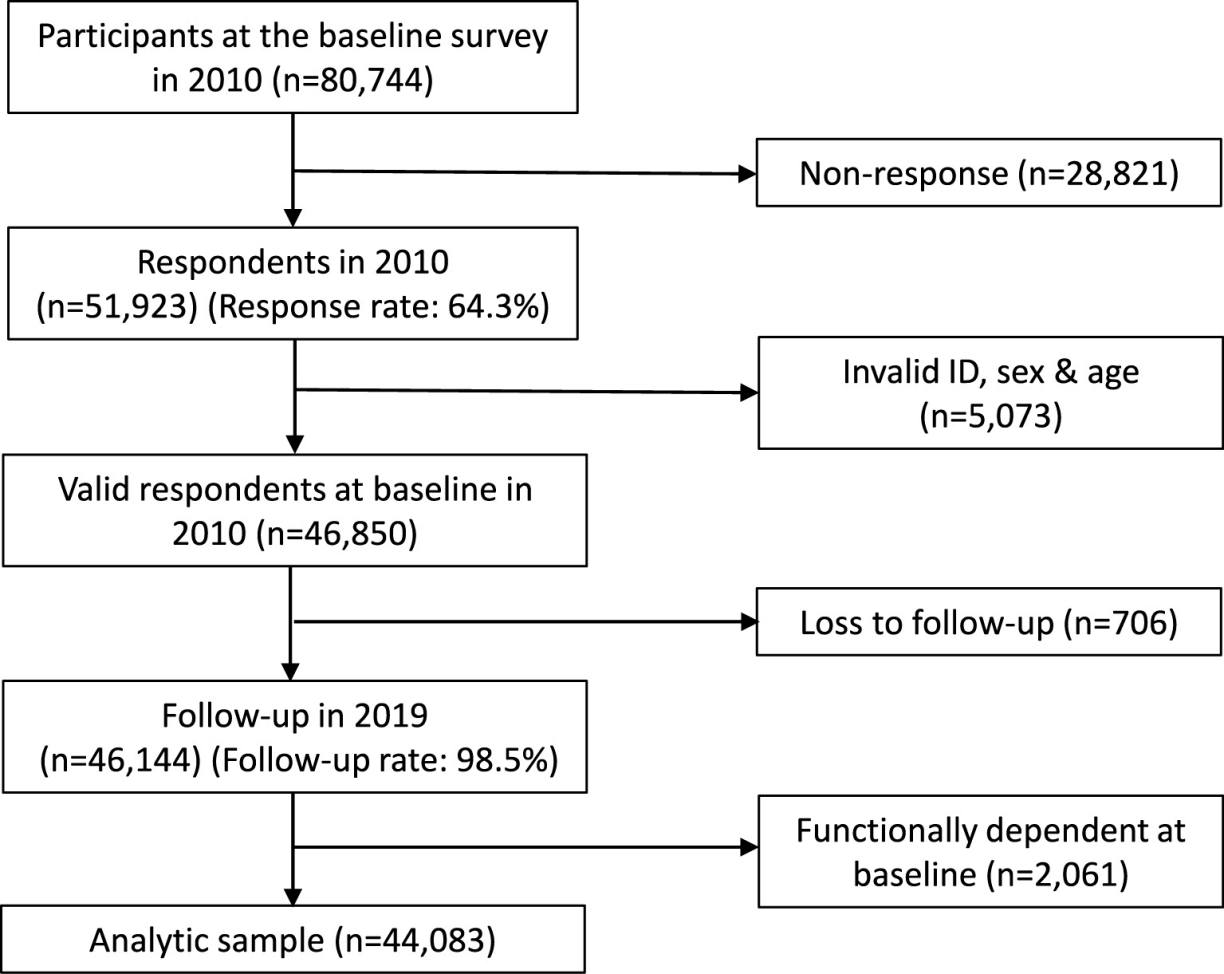
Flowchart of the participants for the present study.

This longitudinal study was based on the Japan Gerontological Evaluation Study (JAGES) from 2010 to 2019 (maximum follow-up period: 3,775 days). JAGES is a longitudinal study targeting physically and cognitively independent people aged 65 years or older. In the survey, questionnaires were sent to participants via mail, and informed consent was obtained from all participants. JAGES aimed to investigate the social determinants of health and the social environment. The baseline survey was conducted in eleven Japanese municipalities in 2010 (n = 80,744), and the number of respondents was 51,923 (response rate = 64.3%). Of these, 46,850 participants were included after excluding individuals with invalid ID, sex, and age information. Among those included, 46,144 participants were followed up until 2019 (follow-up rate: 98.5%). In the analyses, we excluded functionally dependent people at baseline in 2010. Therefore, the number of participants analyzed in this study was 44,083. The flowchart of participants in the present study is shown in Figure 1.

### Outcome variables

We used the incidence of dementia from 2010 to 2019 (maximum follow-up period: 3,775 days) as the outcome. Participants who died without developing dementia during the follow-up period were included in the analyses and were treated as censored at death. The number of dementia incidences and censored participants for each year is shown in Supplementary Table S1. We linked the JAGES data to dementia records obtained from the government database of Japan’s public long-term care insurance (LTCI). People who require long-term care and service apply for certification of LTCI benefits at the municipal office. After the applications are submitted, a certification committee dispatches trained investigators to the applicants’ homes to conduct in-home assessments and medical examinations under the LTCI system, and determines the applicants’ eligibility for care ^(15), (16), (17)^. The investigators completed training conducted by the prefectural government or a designated city ^(18)^. Applicants were evaluated for the following statuses: physical function, activities of daily living, cognitive function, mental and behavioral disorders, adaptation to social life, and past medical treatment were assessed using a standardized protocol ^(15), (16), (17)^. The applicants were classified into 8 levels, according to the severity of cognitive impairment, following the assessments. This classification showed a strong association with the Mini-Mental State Examination (Spearman rank correlation p = −0.74) and was therefore considered valid ^(19)^. Applicants who manifested symptoms that adversely affected daily life or activities were classified as level II (corresponding to a 16-point score on the Mini-Mental State Examination ^(19)^) or higher, and they were defined as having dementia ^(15), (16), (17)^. Additionally, the definition of dementia incidence as classification at level II or higher in cognitive impairment severity was validated in a previous study ^(20)^. The time of dementia incidence was defined as the time when applicants registered for LTCI. We performed causal mediation analyses with a 6-year follow-up as sensitivity analyses (Supplementary Table S2).

### Explanatory variables

We used the equivalent income at baseline (2010) as an explanatory variable, i.e., an indicator of SES. Equivalent income was calculated by dividing annual income by the square root of the number of people in the household. Income was divided into 2 categories, less than 2 million yen and more than 2 million yen, based on criteria that approximately equalized the number of people in each category to apply causal mediation analysis and calculate the mediating effect. Additionally, we conducted mediation analyses that used income as a continuous variable for sensitivity analyses (Supplementary Table S3).

### Mediator

We used smoking status in 2010 as a mediator. Previous studies have reported that smoking is associated with the incidence of dementia and that smoking is one of the main modifiable risk factors for dementia ^(1)^. The participants were categorized in the questionnaire as (1) non-smokers, (2) ex-smokers, and (3) current smokers. In the past, more than 50% of Japanese men smoked ^(21)^, and categorizing ex-smokers as smokers weakened the association between SES and smoking. Therefore, we dichotomized the smoking status as non-smokers and ex-smokers vs current smokers and used it as a mediator variable. In addition, to evaluate the effect of past smoking, we conducted analyses comparing never-smokers and ex-smokers when excluding current smokers from the dataset. The results of the mediating effect of past smoking on the association between income and dementia are shown in Supplementary Table S4 for sensitivity analyses. Furthermore, we analyzed the mediating effect of behavioral factors such as walking time and alcohol consumption habits to compare the extent of the mediating effect of smoking. In the questionnaire, waking time was categorized into ≥90 minutes, 60-89 minutes, 30-59 minutes, and <30 minutes per day. In the mediation analyses, we dichotomized walking time into <30 minutes per day and ≥30 minutes per day and used it as a proxy for physical activity. Alcohol consumption habits were categorized into not drinking, used to drink, and drinking in the survey. We dichotomized alcohol consumption habits into not drinking and used to drink vs drinking in the mediation analyses.

### Covariates

We used covariates measured in 2010 following to previous studies ^(1), (7), (10), (14), (22), (23), (24), (25), (26)^: age, educational attainment (<6 years/6-9 years/10-12 years/≥13 years), self-rated health (very good/good/poor/very poor), Geriatric Depression Scale (GDS, not depression/suggestive depression/depression), marital status (having partner/not having partner), walking time (a proxy for physical activity, ≥90 minutes/60-89 minutes/30-59 minutes/<30 minutes per day), employment status (have job/retired job/never had job), alcohol consumption habit (not drink/used to drink/drink), and region of residence (eleven municipalities) in 2010. Based on previous studies, health and socioeconomic factors (age, educational attainment, self-rated health, GDS, employment status, and region of residence) were considered to affect income and dementia ^(7), (14), (22), (24)^. Age, educational attainment, and region of residence were considered to affect smoking ^(10), (14)^. Lifestyle factors (marital status, walking time, and alcohol consumption habit) were considered to affect both smoking and dementia ^(1), (23), (25), (26)^.

### Statistical analysis

**Figure 2.**
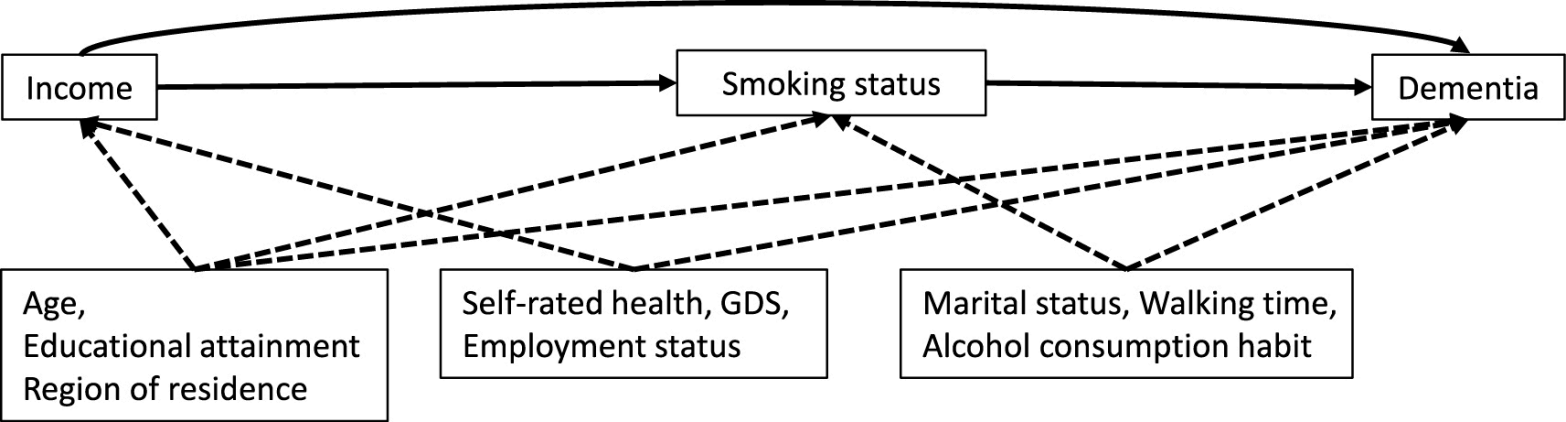
Directed acyclic graph for the present study. ^*^Solid line indicates the effects of exposure, mediator, and outcome. The dashed line indicates the effects of confounders. GDS: Geriatric Depression Scale.

The directed acyclic graph is presented in Figure 2. We performed causal mediation analyses with a Cox proportional hazards model to evaluate the mediating effect of smoking on the association between income and dementia incidence. In the causal mediation analyses with a Cox proportional hazard model, we adjusted for the following covariates: age, educational attainment, self-rated health, GDS, marital status, walking time, employment status, alcohol consumption habit, and region of residence. The Cox proportional hazard model was fitted for outcome regression, while the logistic regression model was fitted for mediator regression. We used the med4way command in Stata to perform causal mediation analyses ^(27)^. We presented the total effect (TE), natural direct effect (NDE) (i.e., the path not through smoking status), natural indirect effect (NIE) (i.e., the path through smoking status), and proportion mediated (PM) ^(28)^. The causal mediation analyses decompose TE into NDE and NIE in a counterfactual framework, and TE, NDE, and NIE were calculated on the excess relative risk (ERR) scale ^(28)^. ERR describes the difference in incremental relative risk and is calculated by subtracting 1 from relative risk. All analyses were stratified by sex because sex differences have been reported in the prevalence of smoking ^(10), (29)^.

The prevalence of missing information on each variable is shown in Supplementary Table S5. We assumed the data were missing at random and performed multiple imputations with chained equations to reduce selection bias due to missing data on the variables ^(30)^ (Supplementary Table S6). Twenty data sets were created by multiple imputations, which included all the variables used in the present study and were combined based on Rubin’s rule ^(30)^. The characteristics of participants in the complete cases are shown in Supplementary Table S7 (n = 28,159). In addition, causal mediation analyses in complete cases were performed for the sensitivity analyses (Supplementary Table S8). All the analyses were performed using Stata MP ^Ⓡ^ 18.0 (Stata Corporation, College Station, TX, USA). The p < 0.05 was considered indicative of statistical significance.

### Ethical considerations

This study was approved by the Ethics Committee of the National Center for Geriatrics and Gerontology (approval number [no.] 1274-2), Chiba University (approval no. 3442), and the Faculty of Dentistry, Institute of Science Tokyo (approval no. D2022-040). It was performed in accordance with the Declaration of Helsinki. We also followed the Strengthening the Reporting of Observational Studies in Epidemiology statement.

## Results

Table 1 shows the characteristic distribution of participants for income by age, sex, educational attainment, self-rated health, smoking status, GDS, marital status, walking time, employment status, and alcohol consumption habit. In this study, 44,083 participants (mean age: 73.7 years, standard deviation = 6.00; 20,634 men and 23,449 women) were included. The age distribution between the high-income and low-income groups was similar. The prevalence of missing information on each variable ranged from 1.1% for self-rated health to 17.0% for equivalent income (Supplementary Table S5). As shown in Supplementary Table S6, imputed samples showed characteristics similar to those of the baseline respondents compared to complete cases.

**Table 1.** Descriptive Distribution of the Participants after Performing Multiple Imputations (N = 44,083).

Characteristic*	Overall participants (N = 44,083)		Men (n = 20,634)		Women (n = 23,449)	
	≥2.0 million JPY	<2.0 million JPY	≥2.0 million JPY	<2.0 million JPY	≥2.0 million JPY	<2.0 million JPY
	n (%)	n (%)	n (%)	n (%)	n (%)	n (%)
Smoking status						
Non-current smoker	19,830 (89.7)	19,351 (88.0)	8,936 (82.1)	7,671 (78.7)	10,894 (97.2)	11,680 (95.5)
Current smoker	2,273 (10.3)	2,629 (12.0)	1,955 (17.9)	2,073 (21.3)	318 (2.8)	556 (4.5)
Age
65-69	7,179 (32.5)	5,885 (26.8)	3,595 (33.0)	2,601 (26.7)	3,584 (32.0)	3,284 (26.8)
70-74	6,439 (29.1)	6,739 (30.7)	3,186 (29.3)	3,055 (31.4)	3,253 (29.0)	3,684 (30.1)
75-79	4,604 (20.8)	5,235 (23.8)	2,227 (20.5)	2,325 (23.9)	2,377 (21.2)	2,911 (23.8)
80-84	2,706 (12.2)	2,813 (12.8)	1,349 (12.4)	1,248 (12.8)	1,357 (12.1)	1,565 (12.8)
≥85	1,175 (5.3)	1,308 (6.0)	533 (4.9)	515 (5.3)	642 (5.7)	793 (6.5)
Educational attainment						
≥13 years	5,584 (25.3)	2,642 (12.0)	3,504 (32.2)	1,508 (15.5)	2,080 (18.6)	1,133 (9.3)
10-12 years	8,935 (40.4)	6,806 (31.0)	4,062 (37.3)	3,010 (30.9)	4,873 (43.5)	3,796 (31.0)
6-9 years	7,310 (33.1)	11,901 (54.1)	3,239 (29.7)	5,028 (51.6)	4,071 (36.3)	6,873 (56.2)
<6 years	274 (1.2)	632 (2.9)	86 (0.8)	197 (2.0)	189 (1.7)	434 (3.5)
Self-rated health						
Very good	2,970 (13.4)	2,357 (10.7)	1,537 (14.1)	1,084 (11.1)	1,433 (12.8)	1,273 (10.4)
Good	15,707 (71.1)	14,889 (67.7)	7,550 (69.3)	6,422 (65.9)	8,157 (72.8)	8,467 (69.2)
Poor	2,989 (13.5)	4,113 (18.7)	1,567 (14.4)	1,898 (19.5)	1,423 (12.7)	2,216 (18.1)
Very poor	437 (2.0)	620 (2.8)	237 (2.2)	339 (3.5)	199 (1.8)	281 (2.3)
GDS						
Not depression	17,331 (78.4)	14,376 (65.4)	8,510 (78.1)	6,261 (64.3)	8,821 (78.7)	8,114 (66.3)
Suggestive depression	3,907 (17.7)	5,496 (25.0)	1,931 (17.7)	2,500 (25.7)	1,976 (17.6)	2,996 (24.5)
Depression	865 (3.9)	2,108 (9.6)	450 (4.1)	982 (10.1)	415 (3.7)	1,127 (9.2)
Marital status						
Having partner	16,558 (74.9)	15,206 (69.2)	9,524 (87.4)	8,323 (85.4)	7,034 (62.7)	6,884 (56.3)
Not having a partner	5,546 (25.1)	6,773 (30.8)	1,367 (12.6)	1,421 (14.6)	4,179 (37.3)	5,353 (43.7)
Walking time						
≥90 minutes	3,647 (16.5)	3,171 (14.4)	1,883 (17.3)	1,506 (15.5)	1,764 (15.7)	1,665 (13.6)
60-89 minutes	3,699 (16.7)	3,281 (14.9)	1,922 (17.7)	1,522 (15.6)	1,777 (15.8)	1,759 (14.4)
30-59 minutes	8,114 (36.7)	7,504 (34.1)	3,973 (36.5)	3,253 (33.4)	4,140 (36.9)	4,251 (34.7)
<30 minutes	6,644 (30.1)	8,024 (36.5)	3,112 (28.6)	3,463 (35.5)	3,532 (31.5)	4,562 (37.3)
Employment status						
Have job	5,779 (26.1)	4,036 (18.4)	3,607 (33.1)	2,214 (22.7)	2,172 (19.4)	1,822 (14.9)
Retired job	13,887 (62.8)	14,449 (65.7)	6,944 (63.8)	6,844 (70.2)	6,943 (61.9)	7,605 (62.2)
Never had job	2,438 (11.0)	3,494 (15.9)	340 (3.1)	685 (7.0)	2,098 (18.7)	2,809 (23.0)
Alcohol consumption habit						
Not drink	12,589 (57.0)	13,988 (63.6)	3,597 (33.0)	3,746 (38.4)	8,992 (80.2)	10,242 (83.7)
Used to drink	717 (3.2)	814 (3.7)	600 (5.5)	657 (6.7)	117 (1.0)	158 (1.3)
Drink	8,797 (39.8)	7,178 (32.7)	6,694 (61.5)	5,341 (54.8)	2,104 (18.8)	1,837 (15.0)

^*^Information about place of residence is omitted as some local governments may consider it to be sensitive information. GDS: Geriatric Depression Scale; JPY: Japanese Yen.

The proportions of current smokers with high income were 10.3% of the overall participants, 17.9% for men, and 2.8% for women. However, the proportions of current smokers with low income were 12.0% of overall participants, 21.3% for men, and 4.5% for women. Therefore, people with low income tended to be current smokers compared to those with high income.

Table 2 shows the incidence rates of dementia (2010-2019) in overall participants and stratified by sex. The incidence rate of dementia in overall participants with high income was 0.020 per person-year, and the incidence rate of dementia in overall participants with low income was 0.025 per person-year. In addition, the incidence rate of dementia in overall participants with non-current smoking status was 0.0225 per person-year, and the incidence rate in those with current smoking status was 0.0235 per person-year. The incidence rates of dementia in men and women showed consistent results. Thus, individuals with low income and current smoking status had higher incidence rates of dementia.

**Table 2.** Incidence Rates of Dementia in Overall Participants and Stratified by Sex (2010-2019).

	Overall participants		Men		Women	
	Dementia incidence (%)	Dementia incidence by person-year	Dementia incidence (%)	Dementia incidence by person-year	Dementia incidence (%)	Dementia incidence by person-year
Total	17.3	0.023	16.2	0.022	18.2	0.023
Equivalent income (JPY)						
≥2.0 million	15.4	0.020	14.4	0.019	16.3	0.021
<2.0 million	19.1	0.025	18.2	0.025	19.8	0.026
Smoking status						
Non-current smoker	17.3	0.0225	16.1	0.022	18.1	0.023
Current smoker	17.1	0.0235	16.7	0.023	19.0	0.026
Age						
65-69	4.8	0.006	5.3	0.006	4.3	0.005
70-74	10.6	0.013	10.9	0.014	10.4	0.013
75-79	23.1	0.031	21.8	0.031	24.1	0.032
80-84	37.2	0.059	33.3	0.054	40.6	0.062
≥85	50.8	0.101	46.0	0.096	54.4	0.105
Educational attainment						
≥13 years	14.4	0.019	14.4	0.019	14.4	0.018
10-12 years	15.2	0.020	13.7	0.018	16.4	0.021
6-9 years	19.1	0.025	18.8	0.026	19.3	0.025
<6 years	39.8	0.065	33.8	0.055	42.5	0.069
Self-rated health						
Very good	11.8	0.015	11.0	0.014	12.7	0.016
Good	15.9	0.020	14.9	0.020	16.7	0.021
Poor	25.5	0.037	24.1	0.036	26.8	0.037
Very poor	28.2	0.048	24.4	0.045	32.7	0.052
GDS						
Not depression	15.4	0.020	14.5	0.019	16.1	0.020
Suggestive depression	21.4	0.029	19.9	0.028	22.8	0.030
Depression	24.2	0.035	22.1	0.033	26.1	0.037
Marital status						
Having partner	14.6	0.019	15.4	0.020	13.6	0.017
Not having partner	24.1	0.033	21.4	0.031	24.8	0.034
Walking time						
≥90 minutes	11.6	0.014	10.2	0.013	13.0	0.016
60-89 minutes	14.2	0.018	13.6	0.018	14.8	0.019
30-59 minutes	16.5	0.021	16.0	0.021	16.9	0.021
<30 minutes	22.1	0.031	20.9	0.030	23.2	0.031
Employment status						
Have job	10.1	0.013	10.1	0.013	10.2	0.012
Retired job	17.9	0.024	17.9	0.024	17.9	0.023
Never had job	26.1	0.036	28.6	0.043	25.5	0.035
Alcohol consumption habit						
Not drink	19.3	0.025	19.1	0.027	19.4	0.025
Used to drink	20.5	0.029	21.5	0.032	16.1	0.021
Drink	13.5	0.017	13.9	0.018	12.4	0.015
Region of residence						
Area 1	16.1	0.021	15.7	0.021	16.4	0.021
Area 2	20.1	0.027	21.0	0.029	19.3	0.025
Area 3	20.5	0.028	19.2	0.027	21.7	0.029
Area 4	22.2	0.028	20.5	0.026	23.6	0.030
Area 5	13.8	0.019	14.3	0.021	13.1	0.018
Area 6	14.9	0.021	14.0	0.020	15.8	0.022
Area 7	17.2	0.024	16.6	0.024	17.7	0.024
Area 8	13.7	0.017	12.8	0.017	14.4	0.018
Area 9	17.7	0.022	15.8	0.020	19.3	0.023
Area 10	15.0	0.017	13.0	0.015	16.9	0.019
Area 11	19.6	0.026	18.2	0.025	20.5	0.027

GDS: Geriatric Depression Scale; JPY: Japanese Yen.

Table 3 shows the results of causal mediation analyses with a Cox proportional hazard model of dementia. The variables included in the mediation analyses are described at the bottom of Table 3. The TE of ERR in low-income was 0.095 (95% confidence interval [CI]: 0.032-0.157) for overall participants, 0.102 (95% CI: 0.011-0.192) for men, and 0.082 (95% CI: −0.003 to 0.168) for women. Smoking mediated the association between income and dementia: the ERR in NIE was 0.007 (95% CI: 0.004-0.011) for overall participants, 0.007 (95% CI: 0.002-0.013) for men, and 0.005 (95% CI: 0.001-0.009) for women. The PM by smoking was 7.7% for overall participants, 7.3% for men, and 6.4% for women. The mediating effects of smoking were higher compared to the mediating effects of physical activity (4.5% in overall participants, 4.9% in men, and 4.5% in women) and alcohol consumption habit (4.2% in overall participants, 5.4% in men, and 3.0% in women).

**Table 3.** Association between Income and Dementia and Mediating Effect of Smoking during 9-Year Follow-Up.

Smoking status			
	Overall participants (N = 44,083)	Men (n = 20,634)	Women (n = 23,449)
	Excess relative risk (95% CI)	Excess relative risk (95% CI)	Excess relative risk (95% CI)
Total effect*	0.095	0.102	0.082
	(0.032-0.157)	(0.011-0.192)	(−0.003 to 0.168)
Natural indirect effect*	0.007	0.007	0.005
	(0.004-0.011)	(0.002-0.013)	(0.001-0.009)
Natural direct effect*	0.087	0.094	0.077
	(0.026-0.149)	(0.005-0.184)	(−0.008 to 0.162)
Proportion mediated (%)*	7.7	7.3	6.4
**Walking time**			
	**Overall participants (n = 44,083)**	**Men (n = 20,634)**	**Women (n = 23,449)**
	**Excess relative risk (95% CI)**	**Excess relative risk (95% CI)**	**Excess relative risk (95% CI)**
Total effect^*^	0.094	0.104	0.082
	(0.032-0.156)	(0.014-0.194)	(−0.003 to 0.167)
Natural indirect effect*	0.004	0.005	0.004
	(0.002-0.007)	(0.001-0.009)	(0.0002-0.007)
Natural direct effect*	0.090	0.099	0.078
	(0.028-0.152)	(0.009-0.189)	(−0.006 to 0.162)
Proportion mediated (%)*	4.5	4.9	4.5
**Alcohol consumption habit**			
	**Overall participants (n = 44,083)**	**Men (n = 20,634)**	**Women (n = 23,449)**
	**Excess relative risk (95% CI)**	**Excess relative risk (95% CI)**	**Excess relative risk (95% CI)**
Total effect*	0.092	0.102	0.078
	(0.030-0.155)	(0.013-0.192)	(−0.007 to 0.164)
Natural indirect effect^*^	0.004	0.006	0.002
	(0.001-0.007)	(0.001-0.010)	(−0.001 to 0.005)
Natural direct effect^*^	0.089	0.097	0.076
	(0.027-0.151)	(0.008-0.186)	(0.009-0.162)
Proportion mediated (%)^*^	4.2	5.4	3.0

CI: confidence interval; GDS: Geriatric Depression Scale. ^*^This analysis included smoking status, age, educational attainment, self-rated health, GDS, marital status, walking time, employment status, alcohol consumption habit, and region of residence.

The causal mediation analyses that used income as a continuous variable (Supplementary Table S3) and complete case analyses (Supplementary Table S8) showed similar results to those of the main analyses. However, the mediating effect of past smoking, based on the analysis excluding current smokers on the association between income and dementia, was not observed (NIE in ERR [95% CI]: 0.0004 [−0.001 to 0.002] for overall participants) (Supplementary Table S4).

## Discussion

This study investigated the mediating effect of smoking on the association between income and the incidence of dementia. Among the study participants, those with lower income had higher incidence rates of dementia than those with higher income. In addition, current smokers had higher incidence rates of dementia than non-current smokers. The results of our mediation analyses among Japanese older adults revealed that smoking mediated 7.7% of the association between low income and the incidence of dementia.

Our results show that the association between income and dementia is in line with previous studies ^(4), (7)^. For example, a previous study conducting a meta-analysis of eleven prospective studies showed a negative association between income and dementia (relative risk [95% CI]: 1.21 [1.04-1.41]) ^(4)^. Our result showed that the ERR of the association between income and dementia was 1.095 in relative risk (0.095 in ERR) in overall participants.

Factors explaining socioeconomic inequalities in dementia are still controversial but are partly explained by a poly-environmental risk score that consists of modifiable risk factors (i.e., coronary heart disease, type-2 diabetes, smoking, etc.) and protective factors (i.e., low-to-moderate alcohol consumption, healthy diet, and high cognitive activity) ^(31)^. A previous study on the poly-environmental risk score in dementia showed that people with the highest wealth had a 52.2% lower risk of dementia than those with the lowest wealth. Meanwhile, the mediating effect of smoking itself has not been well investigated in previous studies; the results of our study suggest a 7.7% mediating effect of smoking on the association between income and dementia, which appears to be a reasonable proportion within the poly-environmental risk.

Moreover, a previous study targeting middle-aged British employees working at London offices between 1985 and 1988 showed the mediating effect of smoking over a 32-year follow-up ^(14)^. In that study, the TE of socioeconomic position on dementia in the hazard ratio was 2.02, and the indirect effect of smoking on the association between socioeconomic position and dementia in the hazard ratio was 1.09 ^(14)^. Our results were in line with the results of the British study; despite our study being conducted on a specific population of older Japanese adults, the results can be considered generalizable to functionally independent older people. Consequently, our results imply the possibility that smoking cessation, even in older age, may lead to decreased inequalities in dementia.

In this study, we categorized ex-smokers in the grouping as non-smokers to evaluate the effect of smoking on the association between income and dementia. Since the history of past smoking may influence the risk of dementia, we analyzed the mediating effect of past smoking by comparing ex-smokers and non-smokers, excluding current smokers (Supplementary Table S4). As a result, we found a minimal mediating effect of past smoking between income and dementia. Consequently, our grouping of ex-smokers as non-smokers appears to have no significant influence on the analysis of the mediating effect of smoking.

In this study, people with low income tended to have low physical activity (Table 1). In addition, people engaging in more physical activity tended to have lower incidence rate of dementia (Table 2). A previous study showed that high income was positively associated with participation in physical activity ^(32)^. Furthermore, physical inactivity is known as one of the modifiable risk factors for dementia ^(1)^. Therefore, our results were consistent with previous studies.

In the aspect of alcohol consumption habit, people who did not drink alcohol had a higher incidence rate of dementia than people who drank alcohol (Table 2). This result is in accordance with a previous study that reported the association between SES and dementia in Japan ^(5)^. A previous systematic review reported that moderate alcohol consumption reduced dementia risk, while heavy alcohol consumption increased dementia risk ^(33)^. Despite the lack of data on the amount of alcohol consumption in this study, the lower risk of dementia among people with drinking habits is probably because many of the participants did not consume alcohol excessively. However, the mediating effect of alcohol consumption habit on the association between low income and dementia was detected in this study, suggesting the possibility that heavy alcohol consumption increases the risk of dementia. This study focused on the mediating effect of smoking, and the presence of alcohol consumption was used to compare their mediating effects. For future research, evaluating dementia risk by the amount of alcohol consumption would be more effective than the drinking habit.

Given the result of the mediation analyses, physical activity and alcohol consumption mediated the association between income and dementia, although the mediating effects were smaller than those of smoking. Thus, physical activity and alcohol consumption would be somewhat effective in decreasing income inequalities in dementia.

Considering the influence of time-related factors on the results of sensitivity analysis for incidence of dementia, we also evaluated a 6-year follow-up between 2010 and 2016 (Supplementary Table S2). The results of the causal mediation analyses for the 6-year follow-up showed that the effect of low income on dementia incidence was weaker than that observed during a 9-year follow-up period (ERR [95% CI]: 0.047 [−0.029 to 0.124] for 6 years, ERR [95% CI]: 0.095 [0.032-0.157] for 9 years in overall participants). However, the effect of current smoking accounted for a greater proportion of mediated dementia incidence than in the 9-year follow-up period (6 years: 17.2%, 9 years: 7.7% in overall participants). Hence, the effect of income on dementia incidence may become stronger over long time periods, while the effect of current smoking on dementia incidence may diminish. However, further studies are needed to determine the time-varying effect of income and smoking on dementia.

There are several possible mechanisms underlying the associations of low income, smoking, and the incidence of dementia. First, low income causes poverty and a lack of material resources, which leads to unhealthy behaviors ^(34)^. Inequalities in access to medical care, including smoking cessation clinics, may adversely affect smoking status, which exacerbates the socioeconomic inequalities of smoking status ^(34)^. Second, people under stress tend to smoke to reduce tension and promote relaxation ^(35)^. In addition, anxiety is significantly associated with the urge to smoke ^(35)^. Thus, there is a possibility that psychological stress and anxiety caused by low income ^(36)^ may accelerate smoking.

Our study revealed that smoking mediated 7.7% of the association between income and the incidence of dementia. The incidence of dementia is increasing more rapidly in low- and middle-income countries than in high-income countries due to aging and the higher presence of modifiable risk factors for dementia ^(26)^. Our study suggests the possibility that smoking cessation and smoking prevention among older people may contribute to reducing health inequalities of dementia worldwide. Strategies such as increasing taxes or prices on tobacco, or providing support for smoking cessation targeting low-income individuals, may be effective in reducing inequalities in dementia ^(11)^ among older people*.*

The present study has several limitations. First, the covariates such as GDS, walking time, and alcohol consumption habit may be influenced by income. If this is the case, the assumption of the mediation analysis that “no exposure-induced mediator outcome confounding” may be violated. As we adjusted for these factors, the TE of income for dementia might be underestimated. Second, we could not measure confounders prior to the baseline survey (2010). Third, we used a self-report questionnaire, which may have led to misclassifications. We considered the misclassifications of the variables to be nondifferential, which would bias the results toward the null and expand the 95% CI ^(37)^. In the present study, significant associations were observed despite this bias. Therefore, the influence of misclassification bias on our results appears negligible. Fourth, dementia incidence might have been underestimated. A previous survey reported that 34.2% of people with dementia were not certified under the Japanese LTCI system ^(38)^. However, of these, three-quarters had mild severity of dementia ^(38)^. Fifth, we could not examine the sex differences in the mediating effect in detail because of the framework of the mediation analyses. In this study, the prevalence of smoking was lower in women than in men (Table 1), and thus we conducted sex-stratified mediation analyses to assess the robustness of the mediating effect of smoking on the association between income and dementia. The results of the mediation analyses in the sex-stratified models showed a similar trend between men and women (Table 3). Sixth, we did not use other SES indicators, such as education and occupation. This study targeted older people aged 65 years or above. Education is usually received when individuals are young and is difficult to modify at older ages ^(3)^. Also, occupation does not typically change in older age. Therefore, this study focused on income as a modifiable SES indicator.

### Conclusions

Smoking partially mediated the association between low income and the incidence of dementia among older Japanese adults. Our results suggest the possibility that smoking cessation and prevention contribute to reducing inequalities in dementia among older adults. Based on this study, further studies assessing the mediating effect of total modifiable risk factors for dementia on the association between SES and dementia in the Japanese population are recommended.

## Article Information

### Conflicts of Interest

None

### Sources of Funding

This study was supported by JST SPRING (JPMJSP2120, JPMJSP2180), Research Institute of Science and Technology for Society (JPMJOP1831) from the Japan Science and Technology Agency (JST), Grant-in-Aid for Scientific Research (20H00557, 21H03153, 21H03196, 22H03299, 22K13558, 23H00449, 23H03117, 23K21500) from JSPS (Japan Society for the Promotion of Science), Health Labour Sciences Research Grants (19FA1012, 19FA2001, 21FA1013, 22FA2001, 22FA1010, 23FA1022) from the Ministry of Health, Labour and Welfare, and Tokyo Medical and Dental University priority research areas grant. The views and opinions expressed in this article are those of the authors and do not necessarily reflect the official policy or position of the respective funding organizations.

### Author Contributions

Satomi Shimada, Yusuke Matsuyama, and Jun Aida designed this study and analyzed the data. Satomi Shimada wrote the initial draft of the present manuscript. Katsunori Kondo contributed to the acquisition of data. Satomi Shimada, Yusuke Matsuyama, Katsunori Kondo, and Jun Aida interpreted the data, and critically reviewed the manuscript. All authors gave final approval and agreed to be accountable for all aspects of this work.

### Approval by Institutional Review Board (IRB)

This study was approved by the Ethics Committee of the National Center for Geriatrics and Gerontology (approval number 1274-2), Chiba University (approval number 3442), and Faculty of Dentistry, Institute of Science Tokyo (approval number D2022-040). This study was performed in accordance with the Declaration of Helsinki. We also followed the Strengthening the Reporting of Observational Studies in Epidemiology statement.

### Disclaimer

Katsunori Kondo is one of the Editors of JMA Journal and on the journal’s Editorial Staff. He was not involved in the editorial evaluation or decision to accept this article for publication at all.

## Supplement

Supplementary Tables
