# Supplementary material for: The Mediating Effect of Smoking on the Association between Income and Dementia among Japanese Older People
Source: JMA J. 2025 Jun 13;8(3):766–76. doi: 10.31662/jmaj.2025-0018 (PMC12329067; doi:10.31662/jmaj.2025-0018)
Supplement: Supplementary Tables [file 2433-3298-8-3-0766-s001.pdf]

Supplementary tables

The Mediating Effect of Smoking on the Association Between Income and Dementia among Japanese Older People

Satomi Shimada[1)], Yusuke Matsuyama[1)], Katsunori Kondo[2),3)], and Jun Aida[1)]

[1)]Department of Dental Public Health, Graduate School of Medical and Dental Sciences, Institute of Science Tokyo, Tokyo, Japan

[2)]Center for Preventive Medical Sciences, Chiba University, Chiba, Japan

[3)]Institute for Health Economics and Policy, Association for Health Economics Research and Social Insurance and Welfare, Tokyo, Japan

List of supplementary tables

Supplementary Table S1. The number of dementia incidences and censored cases each year

Supplementary Table S2. Association between income and dementia and mediating effect of smoking during 6-year follow-up

Supplementary Table S3. Association between income (continuous) and dementia and mediating effect of smoking during 9-year follow-up

Supplementary Table S4. Association between income and dementia and mediating effect of past smoking status during 9-year follow-up when excluding current smokers from the analysis

Supplementary Table S5. Frequency of missing data of the variables

Supplementary Table S6. The distribution of the variables between complete cases and imputed data

Supplementary Table S7. Characteristics of the participants in complete case analysis (n=28,159)

Supplementary Table S8. Association between income and dementia and mediating effect of smoking during 9-year follow-up in complete case analysis

Supplementary Table S1. The number of dementia incidences and censored cases each year

	Dementia incidence	Death incidence	Loss to follow-up
- 1 year	434	751	147
1-2 year	671	1,012	152
2-3 year	735	1,082	164
3-4 year	827	1,071	155
4-5 year	918	1,115	139
5-6 year	805	1,068	94
6-7 year	864	1,047	110
7-8 year	1,252	1,048	146
8-9 year	803	965	88
9- year	296	270	28

Supplementary Table S2. Association between income and dementia and mediating effect of smoking during 6-year follow-up*

	Overall Participants (N=44,083)	Men (n=20,634)	Women (n=23,449)
	Excess relative risk (95%CI)	Excess relative risk (95%CI)	Excess relative risk (95%CI)
Total Effect*	0.047 (-0.029; 0.124)	0.068 (-0.047; 0.182)	0.025 (-0.075; 0.125)
Natural Indirect Effect*	0.008 (0.004; 0.013)	0.008 (0.002; 0.015)	0.006 (0.001; 0.011)
Natural Direct Effect*	0.039 (-0.036; 0.115)	0.059 (-0.054; 0.172)	0.019 (-0.081; 0.118)
Proportion Mediated (%)*	17.2	12.2	25.3

Abbreviation: CI: Confidence Interval.

* Adjusted for age, educational attainment, self-rated health, geriatric depression scale (GDS), marital status, walking time, employment status, alcohol consumption habit, and region of residence.

Supplementary Table S3. Association between income (continuous*) and dementia and mediating effect of smoking during 9-year follow-up**

	Overall Participants (N=44,083)	Men (n=20,634)	Women (n=23,449)
	Excess relative risk (95%CI)	Excess relative risk (95%CI)	Excess relative risk (95%CI)
Total Effect*	0.058 (0.019; 0.098)	0.075 (0.014; 0.136)	0.044 (-0.008; 0.096)
Natural Indirect Effect*	0.004 (0.002; 0.006)	0.005 (0.002; 0.008)	0.002 (0.0005; 0.004)
Natural Direct Effect*	0.054 (0.015; 0.093)	0.070 (0.010; 0.131)	0.041 (-0.010; 0.093)
Proportion Mediated (%)*	7.4	6.6	5.4

Abbreviation: CI: Confidence Interval.

*The results compare the estimates for 1.25 million JPY and 3.18 million JPY. 1.25 million JPY was approximately the 25% percentile of income. 3.18 million JPY was approximately the 75% percentile of income.

**Adjusted for age, educational attainment, self-rated health, geriatric depression scale (GDS), marital status, walking time, employment status, alcohol consumption habit, and region of residence.

Supplementary Table S4. Association between income and dementia and mediating effect of past smoking during 9-year follow-up when excluding current smokers from the analysis*

	Overall participants (N=39,143 – 39,213)*	Men (n=16,587 – 16,623)*	Women (n=22,556 – 22,593)*
	Excess relative risk (95% CI)	Excess relative risk (95% CI)	Excess relative risk (95% CI)
Total Effect*	0.096 (0.029; 0.163)	0.117 (0.017; 0.216)	0.078 (-0.010; 0.165)
Natural Indirect Effect*	0.0004 (-0.001; 0.002)	-0.001 (-0.004; 0.002)	0.001 (-0.001; 0.003)
Natural Direct Effect*	0.096 (0.029; 0.163)	0.118 (0.019; 0.218)	0.076 (-0.011; 0.164)
Proportion Mediated (%)*	0.4	-1.2	1.4

*The current smokers were excluded from the analyses. Therefore, the number of analyzed populations differed between imputed datasets. The analysis was adjusted for age, educational attainment, self-rated health, geriatric depression scale (GDS), marital status, walking time, employment status, alcohol consumption habit, and region of residence.

Abbreviation: CI: confidence interval.

Supplementary Table S5. Frequency of missing data of the variables

Variables	n	%
Age	0	0
Sex	0	0
Region of residence	0	0
Equivalent income	7513	17.0
Smoking status	4212	9.6
Educational attainment	1034	2.3
Self-rated health	474	1.1
GDS	1750	4.0
Marital status	712	1.6
Walking time	2645	6.0
Employment status	5268	12.0
Alcohol consumption habit	2553	5.8

Abbreviation: GDS: Geriatric depression scale

Supplementary Table S6. The distribution of the variables between complete cases and imputed data*

Characteristic*	Baseline respondents n (%)	Complete case n (%)	Imputed data n (%)
Equivalent income			
≥2.0 million JPY	18,531 (50.7)	14,920 (53.0)	22,103 (50.1)
<2.0 million JPY	18,039 (49.3)	13,239 (47.0)	21,980 (49.9)
Smoking status			
Non-current smoker	35,355 (88.7)	24,743 (87.9)	39,181 (88.9)
Current smoker	4,516 (11.3)	3,416 (12.1)	4,902 (11.1)
Age			
65-69	13,064 (29.6)	9,486 (33.7)	13,064 (29.6)
70-74	13,178 (29.9)	8,852 (31.4)	13,178 (29.9)
75-79	9,839 (22.3)	5,739 (20.4)	9,839 (22.3)
80-84	5,519 (12.5)	2,890 (10.3)	5,519 (12.5)
≥85	2,483 (5.6)	1,192 (4.2)	2,483 (5.6)
Educational attainment			
≥13 years	8,070 (18.8)	5,964 (21.2)	8,225 (18.7)
10-12 years	15,408 (35.8)	10,834 (38.5)	15,741 (35.7)
6-9 years	18,695 (43.4)	10,964 (38.9)	19,211 (43.6)
<6 years	876 (2.0)	397 (1.4)	906 (2.1)
Self-rated health			
Very good	5,279 (12.1)	3,680 (13.1)	5,328 (12.1)
Good	30,278 (69.4)	19,766 (70.2)	30,596 (69.4)
Poor	7,010 (16.1)	4,107 (14.6)	7,102 (16.1)
Very poor	1,042 (2.4)	606 (2.2)	1,056 (2.4)
GDS			
Not depression	30,533 (72.1)	20,847 (74.0)	31,707 (71.9)
Suggestive depression	8,970 (21.2)	5,639 (20.0)	9,403 (21.3)
Depression	2,830 (6.7)	1,673 (5.9)	2,973 (6.8)
Marital status			
Having partner	31,320 (72.2)	21,595 (76.7)	31,764 (72.1)
Not having partner	12,051 (27.8)	6,564 (23.3)	12,319 (27.9)
Walking time			
≥90 minutes	6,442 (15.6)	4,494 (16.0)	6,818 (15.5)
60-89 minutes	6,577 (15.9)	4,583 (16.3)	6,980 (15.8)
30-59 minutes	14,686 (35.4)	10,271 (36.5)	15,617 (35.4)
<30 minutes	13,733 (33.1)	8,811 (31.3)	14,668 (33.3)
Employment status			
Have job	9,043 (23.3)	6,777 (24.1)	9,815 (22.3)
Retired job	24,890 (64.1)	18,323 (65.1)	28,336 (64.3)
Never had job	4,882 (12.6)	3,059 (10.9)	5,932 (13.5)
Alcohol consumption habit			
Not drink	25,001 (60.2)	15,533 (55.2)	26,577 (60.3)
Used to drink	1,446 (3.5)	1,055 (3.8)	1,531 (3.5)
Drink	15,083 (36.3)	11,571 (41.1)	15,975 (36.2)

* *Information about place of residence is omitted as some local governments may consider it to be sensitive information.

Abbreviation: GDS: Geriatric depression scale

Supplementary Table S7. Characteristics of the participants in complete case analysis (N=28,159)

Characteristic*	Overall Participants (N=28,159)		Men (n=14,944)		Women (n=13,215)	
	≥2.0 million JPY	<2 million JPY	≥2.0 million JPY	<2 million JPY	≥2.0 million JPY	<2 million JPY
	n (%)	n (%)	n (%)	n (%)	n (%)	n (%)
Smoking status						
Non-current smoker	13,277 (89.0)	11,466 (86.6)	6,693 (82.0)	5,336 (78.7)	6,584 (97.4)	6,130 (95.0)
Current smoker	1,643 (11.0)	1,773 (13.4)	1,468 (18.0)	1,447 (21.3)	175 (2.6)	326 (5.0)
Age						
65-69	5,378 (36.0)	4,108 (31.0)	2,892 (35.4)	1,997 (29.4)	2,486 (36.8)	2,111 (32.7)
70-74	4,497 (30.1)	4,355 (32.9)	2,445 (30.0)	2,254 (33.2)	2,052 (30.4)	2,101 (32.5)
75-79	2,859 (19.2)	2,880 (21.8)	1,602 (19.6)	1,524 (22.5)	1,257 (18.6)	1,356 (21.0)
80-84	1,566 (10.5)	1,324 (10.0)	900 (11.0)	737 (10.9)	666 (9.9)	587 (9.1)
≥85	620 (4.2)	572 (4.3)	322 (3.9)	271 (4.0)	298 (4.4)	301 (4.7)
Educational attainment						
≥13 years	4,137 (27.7)	1,827 (13.8)	2,785 (34.1)	1,152 (17.0)	1,352 (20.0)	675 (10.5)
10-12 years	6,316 (42.3)	4,518 (34.1)	3,162 (38.7)	2,252 (33.2)	3,154 (46.7)	2,266 (35.1)
6-9 years	4,341 (29.1)	6,623 (50.0)	2,174 (26.6)	3,289 (48.5)	2,167 (32.1)	3,334 (51.6)
<6 years	126 (0.8)	271 (2.0)	40 (0.5)	90 (1.3)	86 (1.3)	181 (2.8)
Self-rated health						
Very good	2,146 (14.4)	1,534 (11.6)	1,208 (14.8)	798 (11.8)	938 (13.9)	736 (11.4)
Good	10,672 (71.5)	9,094 (68.7)	5,711 (70.0)	4,545 (67.0)	4,961 (73.4)	4,549 (70.5)
Poor	1,838 (12.3)	2,269 (17.1)	1,081 (13.2)	1,230 (18.1)	757 (11.2)	1,039 (16.1)
Very poor	264 (1.8)	342 (2.6)	161 (2.0)	210 (3.1)	103 (1.5)	132 (2.0)
GDS						
Not depression	11,933 (80.0)	8,914 (67.3)	6,495 (79.6)	4,483 (66.1)	5,438 (80.5)	4,431 (68.6)
Suggestive depression	2,486 (16.7)	3,153 (23.8)	1,373 (16.8)	1,668 (24.6)	1,113 (16.5)	1,485 (23.0)
Depression	501 (3.4)	1,172 (8.9)	293 (3.6)	632 (9.3)	208 (3.1)	540 (8.4)
Marital status						
Having partner	11,764 (78.8)	9,831 (74.3)	7,246 (88.8)	5,900 (87.0)	4,518 (66.8)	3,931 (60.9)
Not having partner	3,156 (21.2)	3,408 (25.7)	915 (11.2)	883 (13.0)	2,241 (33.2)	2,525 (39.1)
Walking time						
≥90 minutes	2,486 (16.7)	2,008 (15.2)	1,407 (17.2)	1,078 (15.9)	1,079 (16.0)	930 (14.4)
60-89 minutes	2,580 (17.3)	2,003 (15.1)	1,486 (18.2)	1,068 (15.7)	1,094 (16.2)	935 (14.5)
30-59 minutes	5,609 (37.6)	4,662 (35.2)	3,036 (37.2)	2,327 (34.3)	2,573 (38.1)	2,335 (36.2)
<30 minutes	4,245 (28.5)	4,566 (34.5)	2,232 (27.3)	2,310 (34.1)	2,013 (29.8)	2,256 (34.9)
Employment status						
Have job	4,071 (27.3)	2,706 (20.4)	2,723 (33.4)	1,594 (23.5)	1,348 (19.9)	1,112 (17.2)
Retired job	9,487 (63.6)	8,836 (66.7)	5,237 (64.2)	4,788 (70.6)	4,250 (62.9)	4,048 (62.7)
Never had job	1,362 (9.1)	1,697 (12.8)	201 (2.5)	401 (5.9)	1,161 (17.2)	1,296 (20.1)
Alcohol consumption habit						
Not drink	7,835 (52.5)	7,698 (58.1)	2,573 (31.5)	2,473 (36.5)	5,262 (77.9)	5,225 (80.9)
Used to drink	507 (3.4)	548 (4.1)	451 (5.5)	464 (6.8)	56 (0.8)	84 (1.3)
Drink	6,578 (44.1)	4,993 (37.7)	5,137 (62.9)	3,846 (56.7)	1,441 (21.3)	1,147 (17.8)

* Information about place of residence is omitted as some local governments may consider it to be sensitive information.

Abbreviation: GDS: Geriatric depression scale.

Supplementary Table S8. Association between income and dementia and mediating effect of smoking during 9-year follow-up in complete case analysis*

	Overall Participants (N=28,159)	Men (n=14,944)	Women (n=13,215)
	Excess relative risk (95% CI)	Excess relative risk (95% CI)	Excess relative risk (95% CI)
Total effect*	0.130 (0.056; 0.203)	0.137 (0.033; 0.242)	0.111 (0.008; 0.215)
Natural indirect effect*	0.012 (0.006; 0.018)	0.011 (0.004; 0.019)	0.008 (0.001; 0.015)
Natural direct effect*	0.118 (0.045; 0.190)	0.126 (0.023; 0.229)	0.103 (0.001; 0.206)
Proportion mediated (%)*	9.3	8.4	7.2

Abbreviation: CI: Confidence interval.

*Adjusted for age, educational attainment, self-rated health, geriatric depression scale (GDS), marital status, walking time, employment status, alcohol consumption habit, and region of residence.
